# Transformation of Agro-Waste into Value-Added Bioproducts and Bioactive Compounds: Micro/Nano Formulations and Application in the Agri-Food-Pharma Sector

**DOI:** 10.3390/bioengineering10020152

**Published:** 2023-01-23

**Authors:** Saroj Bala, Diksha Garg, Kandi Sridhar, Baskaran Stephen Inbaraj, Ranjan Singh, Srinivasulu Kamma, Manikant Tripathi, Minaxi Sharma

**Affiliations:** 1 Department of Microbiology, Punjab Agricultural University, Ludhiana 141004, India; 2 Department of Food Technology, Koneru Lakshmaiah Education Foundation Deemed to be University, Vaddeswaram 522502, India; 3 Department of Food Science, Fu Jen Catholic University, New Taipei City 242062, Taiwan; 4 Department of Microbiology, Dr. Rammanohar Lohia Avadh University, Ayodhya 224001, India; 5 Biotechnology Program, Dr. Rammanohar Lohia Avadh University, Ayodhya 224001, India; 6 Haute Ecole Provinciale de Hainaut-Condorcet, 7800 Ath, Belgium

**Keywords:** agro-waste, valorization, value-added bioproducts, bioactive compounds, micro/nano encapsulation, agri-food-pharma applications

## Abstract

The agricultural sector generates a significant amount of waste, the majority of which is not productively used and is becoming a danger to both world health and the environment. Because of the promising relevance of agro-residues in the agri-food-pharma sectors, various bioproducts and novel biologically active molecules are produced through valorization techniques. Valorization of agro-wastes involves physical, chemical, and biological, including green, pretreatment methods. Bioactives and bioproducts development from agro-wastes has been widely researched in recent years. Nanocapsules are now used to increase the efficacy of bioactive molecules in food applications. This review addresses various agri-waste valorization methods, value-added bioproducts, the recovery of bioactive compounds, and their uses. Moreover, it also covers the present status of bioactive micro- and nanoencapsulation strategies and their applications.

## 1. Introduction

The disposal of agricultural waste is one of the major environmental issues, which poses adverse effects on ecosystems due to the careless dumping of agricultural waste into the environment. Most reports say that untreated and underutilized agro-industrial waste is disposed of by burning, dumping, or placing it in a landfill [1,2]. Untreated garbage contributes to several greenhouse gas emissions, which in turn exacerbate climate change in a variety of ways. In addition to negative effects on the climate, this also results in the release of additional, undesirable gaseous byproducts [3]. Therefore, significant interventions are required for the sustainable use of agro-waste. This can take the form of the development of sustainable energy technologies and the creation of value-added bioproducts. A larger strain on the environment has resulted from increased global output of wastes, with detrimental effects on soil, air, and water resources [4], which in turn threaten the health of populations and the long-term viability of ecosystems. About 21–37% of greenhouse gases are produced by the agricultural sector [5]. This new reality has prompted a model of sustainable development in recent years that calls for substantial shifts in conventional agricultural production methods and waste utilization.

High concentrations of complex carbohydrates, proteins, fibers, polyphenolic components, bioactive compounds, etc., are found in agro-wastes [6]. Despite the fact that organic compound wastes pose a threat to the environment, they could be used as a raw material in a wide range of agricultural, food, and pharmaceutical goods [7]. The agro-residues are not considered a waste because of their high nutrient content; rather, they are used as a source material for new products. Effective pretreatment technologies for agro-waste biomass, its biochemical characteristics, and advanced conversion processes can improve the cost-effectiveness of conversion processes for bioproducts development [8]. Microbial biotechnology and nanotechnology play a pivotal role in the bioconversion of agricultural waste into enzymes and other bioactive compounds in the pharmaceutical sector, vermicompost, organic fertilizers, and biofuels in the agriculture sector, and nutraceuticals and food products [9,10,11,12,13]. Due to its many potential applications, nanotechnology is increasingly being explored in the food and healthcare industries. Due to the aforementioned enhancements in bioavailability and levels of bioactive compounds, the capability for guided administration of bioactive compounds to specific tissues or organs is also boosted. Nanostructured materials have the potential to revolutionize the food business because of their unique features and large surface area. While the potential benefits of nanotechnology to society as a whole have been widely recognized, recently, the field of food science has begun to explore its applications [14]. Using a matrix or inert material, nanoencapsulation is a method for keeping coated substances (food or flavor molecules/ingredients) that are in a liquid, solid, or gaseous form. Stabilizing bioactive compounds via nanoencapsulation allows for more precise control over their release at physiologically active locations [15]. Therefore, this review addresses the valorization of agro-waste into value-added bioproducts, bioactive compounds, and their applications in the agri-energy-pharma sector, along with the present status of bioactive micro/nanoencapsulation strategies and their applications, with the ultimate goal of reducing waste for a sustainable and green environment. In this review, we retrieved 93 articles on agriculture waste, valorization, pretreatment methods, value-added products, and micro- and nanoencapsulation from the Web of Science^TM^ Core Collection.

## 2. Availability of Agro-Waste

There are various sorts of agro-waste in the environment, depending on their origin and availability [16]. Agro-waste consists primarily of cellulose, hemicellulose, and lignin. Due to their structural complexity, lignocelluloses are difficult to break [17]. Various types of wastes, including crop residue, aquaculture waste, and other wastes of agricultural origin, have been reported [18,19]. The two largest available categories of waste are crop residue and agro-industrial waste, both of which are produced in huge quantities every day in the agricultural and food processing industries [20]. On the other hand, the absence of appropriate management techniques for these wastes, which may be traced back to a lack of or limited access to adequate information, has steadily become a significant obstacle that cannot be minimized due to its magnitude.

Because the manure from cattle is a major source of noxious gases, harmful microorganisms, and odor, there is cause for concern regarding both public health and the environment [21]. Unconsumed feed and undigested substances, both of which are expelled from fish as waste in the form of feces, are the two main contributors to aquaculture solid waste [22]. The possibility for contamination of both surface water and groundwater exists if there is an excessive application of a mixture, an incorrect disposal of agro-residue, undiluted chemicals, or even pesticide containers. Utilizing farm waste in a managed manner can improve irrigation and erosion management [23]. The distinction between agricultural residue and other solid fuels, such as charcoal, wood, and charcoal briquettes, is based on their availability and distinguishable qualities.

## 3. Valorization Technologies for Agro-Waste

**Figure 1:**
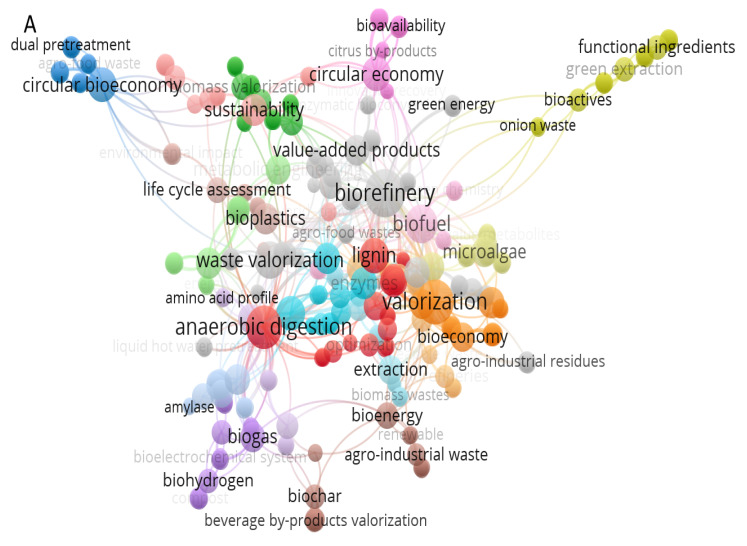

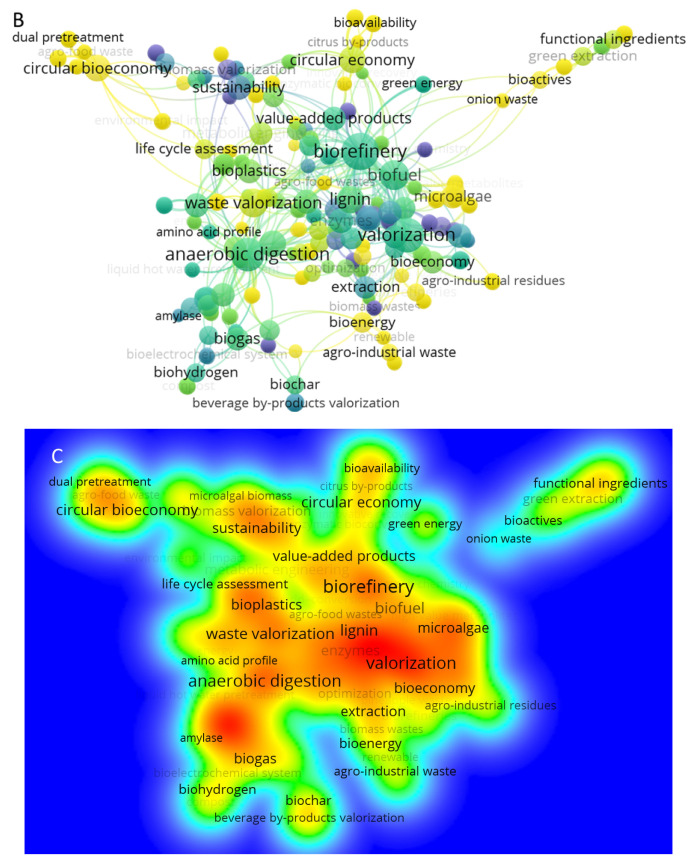
Most frequently used keywords over time, as well as a clustering of the keywords’ citation networks. Network visualization (**A**), overlay visualization (**B**), and density visualization (**C**).

Agro-waste cannot be utilized without a proper pretreatment since modified agro-waste is more effective and useful [24]. Agricultural waste undergoes multiple treatment procedures prior to being utilized. Residues of agro-waste are made of lignocellulosic materials that require chemical, physical, and biological treatments to break the complexity [25]. The transformation of agro-waste’s complex molecular structures into simpler monomers is typically regarded as a necessary pretreatment step [26]. Table 1 details the pros and cons of various types of pretreatment procedures. For better understanding of agro-waste utilization, the graphical mapping of the keyword co-occurrence and co-authorship was constructed by the noncommercial visualization of similarities (VOS) viewer VOS viewer 1.6.18 (https://www.vosviewer.com/, accessed on 20 November 2022). The volume of published work is a strong predictor of future research directions. A keyword list can be used to categorize various fields of study. Figure 1 depicts network visualization, density visualization, and overlay visualization maps of the most-cited keywords from 93 articles. The majority of current research is focused on the development of new value-added products, and the valorization techniques suggest that agricultural waste is among one of the most explored domains that have applications in the agri-food-pharma sector.

**Figure 2:**
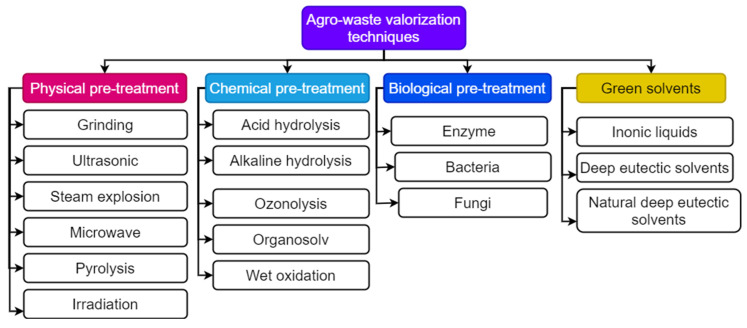
Agro-waste valorization methods for the development of value-added bioproducts.

Wheat straw biodegradation kinetics are increased by mechanical, thermal, and sonic pretreatments but at the expense of high energy consumption [41]. This method decreases the size of biomass and increases its accessible surface area, which improves hydrolysis as a result of enhanced heat and mass transmission [42]. The internal linkages of lignin and hemicellulose are broken due to chemical pretreatment (e.g., the use of acids, alkalis, ozonolysis, organosolv, and wet oxidation) [43]. Pretreatment decreased cassava bagasse weight and boosted cellulose yield significantly compared to unpretreated bagasse. The maximum cellulose yield was achieved with the H_2_SO_4_ pretreatment [44]. The biological process (using microbes) includes hydrolysis and saccharification, which are mediated by exoenzymes. Fermentation using whole-cell systems or simply enzymatic digestion of a solid or liquid substrate is required. This procedure uses less energy, is eco-friendly, and produces no inhibitors [45]. Green pretreatment involves ionic liquids (ILs), deep eutectic solvents, and natural deep eutectic solvents. Pretreatment with ILs improved glucose production from enzymatic hydrolysis by increasing cellulose crystallinity and decreasing particle size and lignin level [46]. Enhanced lignin and cellulose breakdown occurred by breaking intramolecular and intermolecular hydrogen bonds [47]. These pretreatment approaches facilitate enzyme accessibility for hydrolysis, increasing surface area while decreasing operational expenses. Pathak et al. [12] discussed the strategies for the valorization of fruit wastes into value-added compounds. The possible valorization approaches for agro-waste conversion into useful bioproducts and biochemicals are shown in Figure 2.

## 4. Bioactive Compounds from Agro-Waste

In the scientific literature, bioactive compounds (BCs) are described as a natural compound capable of interacting with one or more components of living tissues and exerting a variety of effects [48,49]. In addition, the distinctions between the definitions of dietary supplements, nutraceuticals, and functional foods are sometimes misinterpreted. In contrast to dietary supplements and food additives, nutraceuticals and functional foods are also important [50]. In a nutshell, functional foods contain bioactives that may be helpful to health at higher levels compared to regular foods. These substances may contribute for the better health benefits, as they are deemed superior to conventional foods. Food additives are substances added during food processing to improve food quality and its shelf life [51]. Due to consumers’ increased awareness of the health-promoting effects of nutraceuticals and dietary supplements, interest in adding functional and natural food additives has increased dramatically in recent years. Similarly, consumer interest in health and wellbeing has propelled the expansion of the dietary supplement and nutraceutical markets. Nutraceuticals and dietary supplements can refer to a variety of products with health benefits [52]. However, nutraceuticals are a distinct subset of dietary supplements because they contain pharmaceutical-grade substances but are exempt from the same testing requirements as medicines.

The interest in BCs for various culinary applications continues to expand, fueled by ongoing research efforts to determine the health qualities and prospective applications of these chemicals, which are primarily taken from natural sources, as well as public and consumer interest. Due to their pharmacological properties, they were commercialized as medications and derived mostly from plants, vegetables, and microorganisms [53].

## 5. Application of Agro-Waste in Agri-Food-Pharma

**Figure 3:**
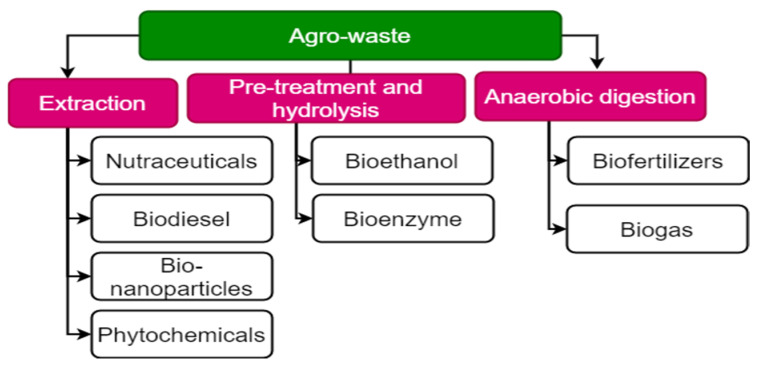
Agro-waste-derived bioproducts and bioactive compounds.

The biobased concept is the primary focus of current research because of its great potential to improve efficiency, cost, and yield, which support and protect environmental sustainability [54]. There are several valuable bioproducts that can be withdrawn from agricultural waste biomass using potential bioconversion pretreatment approaches, such as in the agriculture sector (vermicomposting, biofertilizers, biochar, wood vinegar), the bioenergy sector (biofuels), and the pharma sector (antimicrobials, antioxidants, and antibiotic production). There are various types of bioactives and bioproducts that can be developed from agro-waste, as shown in Figure 3. Agricultural wastes, as mentioned below, can be biotransformed into high-value-added products. 

### 5.1. Agriculture Sector

#### 5.1.1. Vermicomposting

Vermicomposting is the decomposition of organic household waste, and can be used in conjunction with inexpensive, space-saving models to improve soil fertility. Waste items from the agricultural industry that have not been reused or repurposed include both agricultural and processing byproducts [55]. These agri-horticultural wastes are highly biodegradable, making them a major issue in municipal landfills. These are the untapped raw materials with industrial applications [56]. Straw, leaves, twigs, stubbles, and vast quantities of grasses and weeds have all been produced during production of crops and agriculture. By working together, earthworms and aerobic microorganisms stabilize organic waste during the vermicomposting process. Vermicomposting is a low-cost and environmentally friendly method for dealing with agricultural waste. One of the most prevalent earthworm species utilized in vermicomposting is *Eisenia foetida* [57]. Results from physicochemical analyses have indicated that, in comparison to compost and other agricultural wastes, vermicomposting reduces total organic carbon (TOC) and the carbon–nitrogen (C/N) ratio while increasing nitrogen–phosphorus–potassium (NPK) content [58]. Additionally, it can be used to increase agricultural yields by eliminating harmful organisms in the environment (biocontrol), enhancing soil water retention, and manufacturing plant growth regulators. In an effort to revitalize depleted soils, Roohi et al. [59] fertilized maize with either bacteria or compost, or with both. Compost was found to increase both microbial activity and diversity in degraded irrigated soils. Das and Deka [60] demonstrated that nitrogen, phosphorus, and potassium concentrations in compost made from cow dung using *E. fetida* were significantly higher than in compost made from other types of waste. In addition, studies have found that certain species of earthworms secrete the decomposition-promoting phosphatase enzyme [61]. Therefore, vermicomposting can function as a tool for environmental conservation.

#### 5.1.2. Biofertilizers

Soil biofertilizers, which can be made from microorganisms such as bacteria, cyanobacteria, fungi, and algae, are defined as those that improve soil quality by adding nutrients and carbon substrates. Green manures such as cyanobacterial supplements and bioformulations of bacteria such as *Azotobacter* sp., *Azospirillum* sp., *Trichoderma* sp., and arbuscular mycorrhizal fungus (AMF) are the most prevalent and widely used biofertilizers. In addition to microbial “biofertilizers”, farmers frequently employ organic-based fertilizers, including vermicomposting residues, crop residues, and farmyard manure [62,63]. Efforts have been made to develop biofertilizers from agri-waste in the form of discarded melons, pineapples, oranges, bananas, and papayas. Biofertilizer was made using a solid-state fermentation technique and then employed in a vegetable garden. The physical properties of plant samples treated with biofertilizer made from watermelon, papaya, and banana wastes were positively represented in the experimental results. The maximum potassium concentration was found in the banana biofertilizer [64]. Composting and anaerobic digestion (AD) are the two major processes that make use of the metabolic capabilities of the thermophilic and decomposer microbial populations [65]. Enzymes found in native microbial communities aid in bioprocesses for the production of biofertilizers from agro-wastes. 

#### 5.1.3. Bioenergy (Biofuels)

Fuels made from renewable organic biomass, or biofuels, are considered a way to lessen reliance on fossil fuels, lower greenhouse gas (GHG) emissions, particularly from the transportation sector, and increase fuel supply security. Biofuel production from lignocellulosic materials and other wastes is reported by several researchers [10,11,12,13,66]. Enzymes are used to separate monosaccharides from polysaccharides after physicochemical preparation. Enzymes that break down cellulose, hemicellulose, and lignin are required for maximizing sugar monomer release. It has been noted that thermochemical pretreatment releases oligosaccharides that are not digestible by most of the relevant fermentation organisms [13,67]. The use of lignocellulose-derived sugars in ethanol synthesis has received the greatest attention.

Ethanol can be efficiently produced from glucose and other hexoses by using the yeast Saccharomyces cerevisiae, which is the most widely used organism for ethanol production. Brettanomyces bruxellensis and Zymomonas mobilis are yeasts and bacteria, respectively, that can produce ethanol in industrial settings. For example, B. bruxellensis has been proven to ferment oat straw hydrolysate to ethanol [68]. When using straw for biofuels, methane (biogas) production is an additional choice. Energy efficiency, greenhouse gas emissions, and biomass conversion are all improved when switching from ethanol production to biogas production via anaerobic digestion [69]. Compared to ethanol, butanol (both n-butanol and iso-butanol) offers superior fuel properties due to its higher energy density, lower corrosiveness, and greater compatibility with currently used engines. The use of solventogenic Clostridium has enabled the large-scale manufacture of acetone, butanol, and ethanol from starchy materials [70]. The potential of straw or agro-waste as a raw material can be used to manufacture biofuels for environmental sustainability.

### 5.2. Food Sector

**Figure 4:**
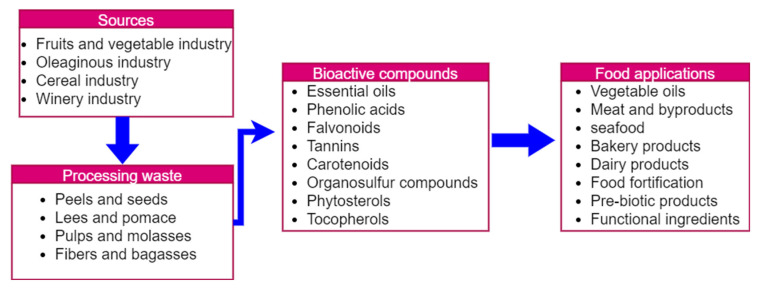
Agro-waste as natural sources of bioactive compounds for food applications. Reprinted from Shirahigue et al. [72] and is an open-access article (copyright © 2023 by authors) distributed under the terms and conditions of the Creative Commons Attribution (CC BY) license.

Agro-waste is a good source of bioactive compounds for food applications. Bioactive compounds isolated from agricultural wastes or byproducts mainly include polyphenolic compounds, vitamins, minerals, fatty acids, volatiles, anthocyanins, and pigments that have valuable health benefits. For example, extracted bioactive compounds can be used in food fortification, such as the development of novel functional and health foods (Figure 4). The great diversity of different bioactive compounds from agro-waste can be a source of natural antioxidants and antimicrobials for wider application in the food and drug industries. Bioactive compounds are widely used in the meat processing industry to prevent lipid oxidation in meat products. In addition, a wider variety of micro- and nanoencapsulation technologies are used to encapsulate these bioactive compounds to increase their applications in the food sector. A study by Kaur et al. [71] extracted the betalains from red beetroot (*Beta vulgaris* L.) pomace and developed a functional Kulfi fortified with encapsulated betalains. This study improved the bioavailability, stability, and solubility of bioactive compounds (betalains) for wider application in the development of dairy-based food products. Likewise, different technologies are being used to improve the applicability of agro-waste-based bioactive compounds in the food sector.

### 5.3. Pharma Sector

#### 5.3.1. Antibiotic Production

Antibiotics are chemicals produced by certain microbes that inhibit or kill the growth of other germs at extremely low doses. Various agricultural byproducts are utilized in the synthesis of various antibiotics [1]. Several research projects employing agro-industrial waste to manufacture antibiotics were conducted. Asagbra et al. [73] investigated antibiotics out of a variety of agricultural byproducts and waste products. When it came to the generation of tetracycline, peanut shells proved to be the most productive substrate at 4.36 mg/g, followed by corncobs. The production of antibiotics was made far more affordable by the utilization of low-cost carbon sources derived from a wide variety of agricultural waste. These residues present an excellent opportunity for innovation in the manufacturing of neomycin and various other antibiotics. In the study conducted by Vastrad and Neelagund [74], the synthesis of extracellular rifamycin B was studied through the use of solid-state fermentation with oil-pressed cake, which is regarded as an agro-industrial waste. An investigation was made into the process of solid-state fermentation used by *Streptomyces speibonae* OXS1 to create oxytetracycline from cocoyam peels, which are considered to be household kitchen wastes of agricultural output [75].

#### 5.3.2. Antioxidant Properties

Antioxidant and anticancer drugs were also developed using agro-waste as a substrate. The remains of various fruits and vegetables, such as fruit and vegetable peels, are usually referred to as waste or useless. Duda-Chodak and Tarko [76] studied the antioxidant capabilities of fruit seeds and peels. In their investigation, they discovered that the peels of selected fruits have the highest antioxidant activity and polyphenol concentration. Pomegranate byproducts from farming produce copious amounts of garbage. It contains ellagitannins, punicalagin, and punicalin, which are extremely powerful antioxidants [77]. Based on their broad range of bioactivities, natural products are often viewed as promising candidates for further value-added research. Phenolic chemicals play a particularly important role among them because of the widespread knowledge of their positive effects on human health, including their function in cancer and cardiovascular disease prevention [78]. Their ability to operate as powerful antioxidants, which are produced in oxidative stress situations and are responsible for the start of a number of inflammatory and degenerative disorders, has been linked to these effects.

#### 5.3.3. Antibacterial and Anticancer Properties

Waste from the agriculture and food industries has the potential to boost the body’s absorption of a wide variety of pharmaceuticals. These are a fantastic resource for a variety of essential nutrients as well as phytochemical substances that can help contribute to a balanced diet. They are a good source of organic and inorganic compounds, sugars, and many phenolic compounds. These phenolic compounds have therapeutic applications due to their anti-inflammatory, antibacterial, immunomodulatory, and antifungal properties. Additionally, they have antibacterial potential due to the production of many antibiotics by microbes produced through agricultural waste. Numerous studies have been conducted on the bioactive compounds found in agricultural residues [49,53]. From the results of orange and lemon processing, millions of tons of waste are generated annually from the industrial manufacture of orange and lemon juice, and this waste is a rich source of hydroxycinnamic acids and flavonoids, primarily flavanone glycosides, flavanones, and flavone aglycons [79]. As byproducts of potatoes, there is little doubt that potato peels are one of the most widely available vegetable waste products. There are a number of potential uses for their extracts, including in the food industry. Chlorogenic acid is the most abundant phenolic acid in peels [80]. Byproducts of lignocellulosic agriculture, such as wheat straw residues, wheat and rice bran, spent residues of coffee ground nuts, and sawdust, have been widely described as a clean source of phenolic compounds and have the potential to be utilized for application in a variety of industries due to the antioxidant and antimicrobial properties that they possess [79].

## 6. Bioactives from Agro Waste: Micro/Nano Formulation and Food Application

The main difference between microencapsulation and nanoencapsulation is the particle size, which normally ranges from 1 micrometer (m) to 1 mm (mm), and the length of time microencapsulation has been deployed [81]. Microencapsulation has been implemented in nearly all industrial sectors, from agriculture and the environment to household and personal care products [82]. Many nanoencapsulated and microencapsulated technologies rely on particle size and distribution homogeneity to be effective. The use of supercritical carbon dioxide (CO_2_) has demonstrated promising advantages for ensuring the proper design of particle size in both microencapsulated and nanoencapsulated pharmaceuticals, as well as the capacity to manage drug-loading procedures across a variety of temperature, pressure, and flow ratios [83]. As noted previously, one of the most alluring features of nanoencapsulation is its capacity to prevent the degradation of active pharmaceutical ingredients. Nanoencapsulation has also enhanced the accuracy of medication delivery targets by coating or conjugating the surface to enable proper cell entrance [84]. In addition, nanoencapsulated pharmaceuticals can be labeled with fluorescent probes for imaging, which is particularly important for assessing therapeutic efficacy during preclinical and clinical research. In the agriculture sector, nanofertilizers are advantageous for nutrition management due to their high potential for enhancing nutrient utilization efficiency. Alone or in combination, nutrients are bonded to nanodimensional adsorbents, which release nutrients much more slowly than conventional fertilizers.

**Figure 5:**
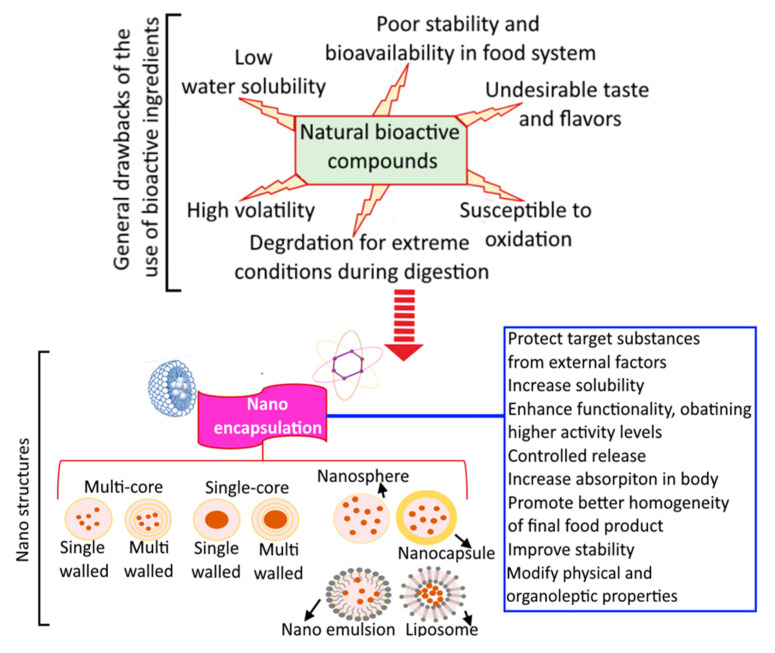
Limitations of bioactive compounds and application of nanoencapsulation to increase bioactive compounds applicability in food industry. Reprinted from Pateiro et al. [15] and is an open-access article (copyright © 2023 by authors) distributed under the terms and conditions of the Creative Commons Attribution (CC BY) license.

There are a number of conventional techniques for microencapsulating food components; however, no single technique is compatible with the core materials or product applications. As previously indicated, the process of microencapsulation is governed by the application and factors such as the target particle size, physicochemical properties of the external and interior phases, release mechanisms, total cost, etc. [85]. As the encapsulated chemicals are often in liquid form, drying techniques are commonly used to convert the liquid phase into a stable powder. To encapsulate active agents of food ingredients and nutraceuticals, various techniques are available [86]. Numerous BACs and other hydrophobic or poorly water-soluble nutrients are crucial for human nutrition and the maintenance of consumer health and wellbeing. Incorporating these compounds into the pharmaceutical and food sectors necessitated overcoming their poor solubility and bioavailability [15]. Nanoencapsulation is a promising strategy for protecting various food elements from certain physiological conditions or deterioration while also disguising some disagreeable aromas and flavors. Reducing the particle size of BACs could improve their stability, bioavailability, solubility, and delivery, hence enhancing their functional activity [87]. In addition, nanoencapsulation can regulate the release of active compounds and improve the final food product’s qualities. Nanocapsulated bioactives and their advantages are presented in Figure 5. 

Advances in nanotechnology have been made in the food science and industry recently. When it comes to protecting the public’s health and enhancing the functional qualities of food formulations, nanoencapsulation is still one of the most promising options. Thus, new methods for encasing bioactive compounds are constantly being introduced, each with their own set of benefits and drawbacks [15]. By using nanoencapsulation, food scientists and engineers can create protective shields that can withstand harsh environments, hide off-putting flavors and odors, and improve nutrient absorption, release, and delivery while also increasing the solubility of lipophilic bioactive substances in water [88]. There is a high probability of overlap between the many uses of nanoencapsulation methods. Therefore, features such as release patterns, safety and toxicity concerns, and economic considerations are taken into account when selecting a nanocarrier for the bioactive molecule.

## 7. Future Prospective and Limitations

Agricultural residues are a potential resource for the synthesis of high-value compounds. In the future, biotechnological methods will be utilized to extract high-value bioproducts from agricultural waste. Because of their low energy requirements and low cost, integrated nano- and biotechnological techniques are preferred for industrial waste valorization. Furthermore, biotechnological methods for agricultural waste valorization are viable solutions for developing unique bioproducts for a variety of industries [54]. Therefore, it is necessary to design eco-friendly and cost-effective cascade conversion techniques. Agricultural waste is seen as a nutritional and functional raw resource that can be utilized in numerous applications, making it a viable solution to economic and environmental issues [89,90]. They can be employed directly as protein, fat, vitamin, fiber, carbohydrate, mineral, and antioxidant dietary components. Other biomolecules from agricultural waste can be recovered physically or chemically and employed as nutritional and functional components. In order to preserve the physicochemical and microbiological stability of bioproducts during the valorization of these agricultural wastes, the unitary drying process is required to prevent microbial hazards [91]. Therefore, governments should promote the installation of infrastructure and technology that enables the use of agricultural leftovers and trash in production and storage regions. The elimination of other dangers, such as poisonous substances and antinutritional factors, must also be considered [92]. Thus, such unique and worthwhile technical implementation may be helpful in addressing such problems across all sectors of agro-waste usage. A well-managed supply chain, supported by analysis, can also help in the disposal of agricultural waste. Agro-industrial food waste can be a useful low-cost feedstock for sustainable production of industrial products [93]. Thus, an appropriate green technology is required for bioproducts development. Moreover, the difficulties of economics and market resistance are reduced over time through effective policies and assessments of their execution.

## 8. Conclusions

Together, environmental policy and the growing demand for a wide variety of biobased products have led to a shift toward a greater focus on finding new uses for agricultural byproducts. Wastes from the food and agriculture industries are rich sources of both nutrients and bioactive compounds. It is more accurate to refer to these materials as “feedstock” rather than “waste” when addressing their possible use in the development of bioproducts due to the fact that their composition might vary, including the presence of sugars, minerals, and proteins. The analysis presented here focuses mostly on agricultural waste, which was valorized in a number of ways to produce value-added products. Valorization through biotechnological and green approaches are helpful in bioproducts development in a cost-effective and comprehensive way. In order to establish a safe and green environment, it is beneficial to recycle wastes from the agricultural and agro-based sectors and use them as feedstock for development of various value-added molecules. Besides these, nanomaterials-based technology can also be used to increase bioactive compounds applicability in various industrial sectors.

## Figures and Tables

**Table 1:** Application based pretreatment methods and related pros as well as cons.

Application	Pretreatment Methods		Pros	Cons	Refs.
**Agriculture sector** Biofuels and manure Enzymatic digestibility Ethanol production Bio-oil and biochar formation **Food sector** Bioactive compounds Nutraceuticals Ethanol and enzyme production	Physical	Grinding	From biomass, a fine powder with a crystallinity of up to 0.2 mm is produced.	Lack of long-term viability in technique calls for a lot of energy.	[27,28,29,30,31,32]
		Ultrasonic	Easing the process of breaking down a variety of lignocellulosic materials.	Collisions between particles during prolonged sonication could result in an antagonistic effect.	
		Steaming explosion	Minimal need for energy.	Incomplete lignin-carbohydrate matrix cleavage, xylan fraction destruction, creation of hydrolysis, and fermentation inhibitors.	
		Microwave	Easily functional, and with efficiency in handling large agro-waste with fewer inhibitors being formed.	This causes both a rise in temperature and an increase in the amount of electricity used.	
		Pyrolysis	The highest possible rate of cellulose sugar conversion.	High-cost technique.	
		Irradiations	The surface area was increased, crystallinity was reduced, hemicelluloses were hydrolyzed, and the structure of lignin was altered.	Expensive method.	
**Pharma sectorSugars** (glucose, xylose, mannose, and galactose) and organic acids (formic, acetic acid) production **Agriculture sector** Enzymes production, organic acids, and hydrolysis of agro-waste to increase glucose yield Biorefinery Biomass saccharification, bioethanol and biogas production **Food sector** Extraction of phenolic compounds and acids productions	Chemical	Acid hydrolysis (HCl, CH_3_COOH, H_2_SO_4_)	Change the structure of lignin, and hydrolyze hemicellulose to xylose and other sugars.	Corrosion of expensive equipment and the production of harmful byproducts are additional costs.	[2,29,33,34,35]
		Alkaline hydrolysis (KOH, NaOH, NH_4_OH, Mg(OH)_2_, Ca(OH)_2_	Pretreatment under milder conditions. Removing lignin and hemicelluloses raises the available surface area.	High alkalinity concentrations and lengthy residence durations are necessary.	
		Ozonolysis	Decreases lignin content. Does not indicate the production of hazardous substances.	Method that is both expensive and demanding of a substantial quantity of ozone.	
		Organosolv	Hydrolyzes lignin and hemicellulose; helpful for lignin extraction.	Due to their high volatility, costly solvents are unsuitable for industrial use.	
		Wet oxidation	Effectively eliminated lignin and low formation inhibitors.	Expensive because of the utilization of oxygen and acid catalyst.	
**Agriculture sector** Animal manure and biofertilizers Biorefinery and animal feed **Pharma sector** Antibiotics production **Food sector** Single cell protein	Biological	Enzyme	Moderate circumstances are present, and minimal effort is necessary.	Low hydrolysis rate and a large sterile space requirement.	[13,23,36,37]
		Bacteria	Economical and requiring only mild reaction conditions.		
		Fungi	Inexpensive, destroys lignin and hemicelluloses, minimal energy needs.		
**Food and pharma sectors** Antibiotic production Antioxidant properties Antibacterial and anticancer properties	Green solvents	Ionic liquids	Effective at dissolving copious amounts of cellulose and recovering usable cellulose from lignin.	Possible toxicity, prohibitively expensive method, and a lack of practicality for mass production.	[38,39,40]
		Deep eutectic solvents	Conditions are modest but environmentally friendly and safe.	Creates undesirable contaminants and higher viscosity on occasion.	
		Natural deep eutectic solvents	Low-cost, readily available, highly modifiable, and less hazardous.		

## Data Availability

Data are available within the article.
